# Effects of blood lipid stability on progression of carotid atherosclerosis

**DOI:** 10.12669/pjms.333.12593

**Published:** 2017

**Authors:** Yu Ding, Bo Li, Feng Tian, Shanshan Zhou, Yundai Chen

**Affiliations:** 1 Yu Ding, Department of Cardiology, Chinese PLA General Hospital, Beijing 100853, China; 2 Bo Li, Department of Cardiology, Chinese PLA General Hospital, Beijing 100853, China; 3 Feng Tian, Department of Cardiology, Chinese PLA General Hospital, Beijing 100853, China; 4 Shanshan Zhou, Department of Cardiology, Chinese PLA General Hospital, Beijing 100853, China; 5 Yundai Chen, Department of Cardiology, Chinese PLA General Hospital, Beijing 100853, China

**Keywords:** Atherosclerosis, Blood lipid, Carotid Artery, Progression

## Abstract

**Objective:**

To evaluate the effects of blood lipid stability on progression of carotid atherosclerosis.

**Methods:**

A total of 416 patients who had physical examination in our hospital annually from January 2010 to December 2015 were selected and divided into a progression group (n=216) and a non-progression group (n=200) according to the intima-media thickness measured by carotid ultrasound. The levels of lipid-related parameters within five years were retrospectively analyzed to calculate the smoothness index (SI = x±/s).

**Results:**

The cross-sectional TG, HDL-C, ApoAI, ApoB, ApoE and Lpa levels were similar in the two groups (p>0.05). The non-progression group had significantly higher TC ((4.15±0.82 vs. 4.50±1.04) mmol/L) and LDL-C ((2.53±0.76 vs. 2.99±1.03) mmol/L) levels than those of the progression group (p<0.05). The progression group had significantly lower TC SI (5.29±1.28 vs. 5.65±1.76), TG SI (2.13±0.71 vs. 2.79±0.82), LDL-C SI (3.66±1.17 vs. 4.36±1.58), ApoB SI (3.37±0.88 vs. 3.62±0.95) and Lpa SI (1.53±0.49 vs. 1.62±0.43) than those of the non-progression group (p<0.05).

**Conclusion:**

Compared with cross-sectional results, SI was better correlated with the progression of atherosclerosis. The progression group had lower SI values.

## INTRODUCTION

Atherosclerosis (AS) has become one of the most dangerous diseases threatening human health, which mostly occurs at the carotid artery.^1^,^2^ The metabolism of lipids and related lipoproteins plays a central role in AS. Therefore, researchers have focused on the levels of LDL-C, HDL-C, TC and TG, mainly aiming to reduce that of LDL-C.^3^,^4^ However, only decreasing LDL-C cannot completely stop AS-induced cardiovascular endpoint events such as death, nonfatal myocardial infarction and nonfatal stroke, probably because the levels of LDL-C, HDL-C, TC and TG do not represent the basic factors of AS. Up to now, whether the fluctuation of levels of lipid-related parameters is related with AS progression remains unclear.In this study, we retrospectively analyzed the relationship between blood lipid stability and progression of carotid AS.

## METHODS

A total of 416 patients who had physical examination in our hospital annually from January 2010 to December 2015 were selected, comprising 410 males (98.56%) and 6 females (1.44%) aged from 50-97 years old, (70.80±12.12) on average. The intima-media thickness (IMT) of carotid artery was measured by color Doppler ultrasound.

### Inclusion criteria

With ethics committee approval and written informed consent; age ≥65 years old; patients who received blood lipid level examination and carotid ultrasound annually. IMT ≥1.0 mm was defined as thickening, and IMT ≥1.5 mm with local eminence of the intima towards the lumen was defined as occurrence of AS.

### Diagnostic criteria for carotid stenosis

Carotid stenosis, <50%; stenosis, 50%-69%; stenosis, ≥70% to near occlusion; complete occlusion.

### Determination criteria for progression of carotid AS

Carotid ultrasound in 2010 disclosed carotid AS, without stenosis, but ultrasound in 2015 showed carotid stenosis; carotid stenosis was aggravated by at least one grade from 2010 to 2015. Progression of carotid AS was defined if any one of the above two situaitons occurred. The selected patients were divided into a progression group (n=216) and a non-progression group (n=200). The progression group consisted of 213 males and 3 females aged from 51 to 97 years old, (71.04±12.36) on average. The non-progression group comprised 197 males and 3 females aged from 50 to 95 years old, (69.49±11.30) on average. Siemens Acuson Sequoia 512 ultrasound system with a probe at the frequency of 13-15 MHz was used.

### Measurement of carotid artery IMT

After 5-10 minutes of rest, a patient in the supine position was first subjected to blood pressure detection of the brachial artery. The head was tilted to one side to entirely expose the neck on the other side. Afterwards, horizontal and vertical scannings for the carotid artery were performed respectively, aiming at 15 mm above the common carotid artery to 15 mm below the bifurcation level. The probe was held perpendicularly to the common carotid artery, and the gain and sampling box were adjusted. Meanwhile, the image was adjusted to clearly show the intima and media at the posterior and anterior walls of the maximum longitudinal section. IMT was continuously and stably measured three times, and the mean was recorded. The examination was conducted by one sonographer according to uniform evaluation criteria.

### General information

The age, gender, body weight, height, systolic pressure, diastolic pressure and ventricular rate were recorded, and the body mass index (BMI) was calculated. Whether the patients had coronary artery disease, hypertension, diabetes mellitus, cerebrovascular disease or history of smoking was inquired.

### Laboratory detection

Fasting venous blood (4 ml) was collected on the early morning of examination. The levels of glycosylated hemoglobin, uric acid, total bilirubin, and direct bilirubin, LDL-C, HDL-C, TC and TG were detected by Roche cobas 8000 modular analyzer. Blood lipid stability was calculated according to the levels and expressed as smoothness index (SI = x±/s, where x± and s represent the mean and standard deviation of blood lipid levels detected 5 times from 2010 to 2015). A high SI means a small fluctuation and a high stability. A low SI means a large fluctuation.

### Statistical analysis

All data were analyzed by SPSS 19.0. The numerical data were compared by the Chi-square test. The categorical data were expressed as mean ± standard deviation. The data conforming to normal distribution between groups were compared by the independent samples t-test, and those abnormally distributed were compared by the non-parametric test. The relationship between normally distributed data was calculated by the Pearson’s correlation analysis. The factors related to carotid atherosclerosis were subjected to univariate and multivariate logistic regression analyses. P<0.05 was considered statistically significant.

## RESULTS

### Baseline clinical data

The two groups had similar age, gender, blood pressure, BMI and disease history (p>0.05) (Table-I).

**Table-I:** Baseline clinical data.

	*Progression group (n=216)*	*Non-progression group (n=200)*	*P*
Age (year)	71.04±12.36	69.49±11.30	0.184
Male [case (%)]	213 (98.59)	197 (98.50)	0.924
Systolic pressure (mmHg)	140.62±31.72	135.69±28.21	0.096
Diastolic pressure (mmHg)	65.87±19.64	67.91±20.83	0.305
BMI	26.26±3.78	25.72±3.49	0.065
History of hypertension [case (%)]	158 (73.15)	161 (80.50)	0.076
History of coronary artery disease [case (%)]	172 (79.63)	162 (81.00)	0.726
History of diabetes mellitus [case (%)]	98 (45.37)	109 (54.50)	0.063
History of cerebrovascular disease [case (%)]	59 (27.31)	60 (30.00)	0.545
History of smoking [case (%)]	99 (45.83)	80 (40.00)	0.230

### Cross-sectional blood lipid levels

The cross-sectional TG, HDL-C, ApoAI, ApoB, ApoE and Lpa levels were similar in the two groups (p>0.05). The non-progression group had significantly higher TC and LDL-C levels than those of the progression group (p<0.05) (Table-II).

**Table-II:** Cross-sectional blood lipid levels.

	*Progression group (n=216)*	*Non-progression group (n=200)*	*P*
TC (mmol/L)	4.15±0.82	4.50±1.04	0.000
TG (mmol/L)	1.30±0.40	1.41±0.84	0.085
LDL-C (mmol/L)	2.53±0.76	2.99±1.03	0.000
HDL-C (mmol/L)	1.33±0.26	1.39±0.42	0.078
ApoAI (mg/dl)	1.28±0.26	1.31±0.32	0.293
ApoB (mg/dl)	1.00±0.31	0.92±0.28	0.080
ApoE (mg/dl)	5.35±2.48	5.58±2.00	0.300
Lpa (mg/dl)	17.47±12.72	19.20±13.17	0.174

### SI values of lipid-related parameters

Blood lipid stability was calculated according to the levels and expressed as smoothness index (SI = x±/s, where x± and s represent the mean and standard deviation of blood lipid levels detected 5 times from 2010 to 2015). The progression group had significantly lower TC SI, TG SI, LDL-C SI, ApoB SI and Lpa SI than those of the non-progression group (p<0.05) (Table-III).

**Table-III:** SI values of lipid-related parameters.

	*Progression group (n=216)*	*Non-progression group (n=200)*	*P*
TC SI	5.29±1.28	5.65±1.76	0.017
TG SI	2.13±0.71	2.79±0.82	0.000
LDL-C SI	3.66±1.17	4.36±1.58	0.000
HDL-C SI	3.45±0.50	3.53±0.86	0.243
ApoAI SI	5.16±1.60	5.26±0.93	0.441
ApoB SI	3.37±0.88	3.62±0.95	0.006
ApoE SI	2.77±1.69	2.99±0.65	0.085
Lpa SI	1.53±0.49	1.62±0.43	0.048

## DISCUSSION

Disorders of blood lipid metabolism have been closely associated with AS. It is well-established that continuous blood lipid abnormalities predominantly promote AS onset and progression. Besides, high LDL-C level indeed mainly leads to AS.^5^,^6^ However, some patients with acute coronary syndrome may not have elevated LDL-C levels.^7^ The main pathological and physiological mechanisms for acute coronary syndrome are rupture of vulnerable AS plaque and thrombosis. Therefore, the factors affecting the progression and stability of AS plaque have attracted wide attention.^8^ An animal study using New Zealand white rabbits^9^ showed that after 24 weeks of feeding, the blood lipid levels were measured regularly to calculate SI. A lower SI suggested faster progression of AS. In this study, the levels of lipid-related parameters within five years were retrospectively analyzed. The cross-sectional TG and HDL-C were similar in the two groups. The non-progression group had significantly higher TC ((4.15±0.82 vs. 4.50±1.04) mmol/L) and LDL-C ((2.53±0.76 vs. 2.99±1.03) mmol/L) levels than those of the progression group. The progression group had significantly lower TC SI, TG and LDL-C SI than those of the non-progression group. SI values of lipid-related parameters affected AS progression more significantly than cross-sectional blood lipid levels did. A lower SI meant a larger fluctuation.

Recently, the levels of apolipoproteins have also been highlighted in studies concerning AS. Apolipoproteins are synthesized mainly in the liver and partly in the small intestine. They have been classified into several subtypes and represented by Roman numerals.^10^,^11^ ApoA mainly contains AI and AII. As a single-chain polypeptide, ApoAI is synthesized in the liver and intestinal mucosa, mainly existing in HDL and being negatively correlated with AS.^12^-^14^ ApoB mainly exists in LDL and chylomicron, which reflects the number of LDL particles.^15^,^16^ Increase of ApoB is an important risk factor for AS.^14^ As a polymorphic protein, ApoE is the ligand of LDL receptor and that of chylomicron particle receptor in hepatocytes. ApoE protects against AS by transporting endogenous and exogenous cholesterol.^17^ Lpa is one of plasma lipoproteins, which is linked by disulfide bonds from a part of LDL and ApoA. It is actually an LDL with a special form that is synthesized in the liver.^18^,^19^ Lpa has been proven to be one of the independent risk factors for AS. It promotes both the inflammatory response and progression of AS.^20^ In this study, the cross-sectional ApoAI, ApoB, ApoE and Lpa levels were similar in the two groups. The progression group had significantly lower ApoB SI and Lpa SI than those of the non-progression group. Compared with solely detecting cross-sectional blood lipid levels, dynamically observing the fluctuations of blood lipid and apolipoprotein levels can better indicate the progression of carotid AS. The findings are of great significance to the regulation of compliance for taking hypolipidemic drugs and the stabilization of blood lipid levels.

### Limitations of the study

Firstly, as a retrospective study, the sample size is small. Secondly, more female cases should be selected to validate the conclusion of this study. Thirdly, the mechanism by which fluctuations of blood lipid levels promote AS progression remains unclear. Further studies are ongoing in our group.

### Authors` Contribution

**YD and YC** designed and performed this study, and prepared this manuscript. **BL, FT and SZ** collected and analyzed related clinical data.

## References

[1] Polak JF, Szklo M, O’Leary DH (2017). Carotid Intima-Media Thickness Score, Positive Coronary Artery Calcium Score, and Incident Coronary Heart Disease: The Multi-Ethnic Study of Atherosclerosis. J Am Heart Assoc.

[2] Selwaness M, Bos D, van den Bouwhuijsen Q, Portegies ML, Ikram MA, Hofman A (2016). Carotid Atherosclerotic Plaque Characteristics on Magnetic Resonance Imaging Relate with History of Stroke and Coronary Heart Disease. Stroke.

[3] Nimkuntod P, Tongdee P (2015). Plasma Low-Density Lipoprotein Cholesterol/High-Density Lipoprotein Cholesterol Concentration Ratio and Early Marker of Carotid Artery Atherosclerosis. J Med Assoc Thai.

[4] Fawwad A, Sabir R, Riaz M, Moin H, Basit A (2016). Measured versus calculated LDL-cholesterol in subjects with Type-2 diabetes. Pak J Med Sci.

[5] Martin SS, Blumenthal RS, Miller M (2012). LDL cholesterol: the lower the better. Med Clin North Am.

[6] Luo KQ, Feng XW, Xu BC, Long HB (2014). Association between arterial stiffness and risk of coronary artery disease. Pak J Med Sci.

[7] Bayturan O, Utuk O, Tuzcu EM (2011). Beyond lowering LDL cholesterol. Anadolu Kardiyol Derg.

[8] Holschermann H, Tillmanns H, Bode C (2006). Pathophysiology of acute coronary syndrome. Hamostaseologie.

[9] Zou X, Wang H, Cai L, Li K, Zhang W, Ding Y (2014). Effects of serum lipid smoothness on the progression and vulnerability of atherosclerotic plaques in rabbits. PloS One.

[10] Teoh CL, Griffin MD, Howlett GJ (2011). Apolipoproteins and amyloid fibril formation in atherosclerosis. Protein Cell.

[11] Seishima M (2016). Physiological Function of Apolipoproteins and Atherosclerosis. Rinsho Byori.

[12] Gordon SM, Davidson WS (2012). Apolipoprotein A-I mimetics and high-density lipoprotein function. Curr Opin Endocrinol Diabetes Obes.

[13] Leman LJ, Maryanoff BE, Ghadiri MR (2014). Molecules that mimic apolipoprotein A-I: potential agents for treating atherosclerosis. J Med Chem.

[14] Sniderman AD, Marcovina SM (2006). Apolipoprotein A1 and B. Clin Lab Med.

[15] Sniderman A, Couture P, de Graaf J (2010). Diagnosis and treatment of apolipoprotein B dyslipoproteinemias. Nat Rev Endocrinol.

[16] Morita SY (2016). Metabolism and Modification of Apolipoprotein B-Containing Lipoproteins Involved in Dyslipidemia and Atherosclerosis. Biol Pharm Bull.

[17] Yousuf FA, Iqbal MP (2015). Review: Apolipoprotein E (Apo E) gene polymorphism and coronary heart disease in Asian populations. Pak J Pharm Sci.

[18] Bucci M, Tana C, Giamberardino MA, Cipollone F (2016). Lp(a) and cardiovascular risk: Investigating the hidden side of the moon. Nutr Metab Cardiovasc Dis.

[19] Manocha A, Srivastava LM (2016). Lipoprotein (a): a Unique Independent Risk Factor for Coronary Artery Disease. Indian J Clin Biochem.

[20] Gouni-Berthold I, Berthold HK (2011). Lipoprotein(a): current perspectives. Curr Vasc Pharmacol.

